# Optimized Configuration of Functional Brain Network for Processing Semantic Audiovisual Stimuli Underlying the Modulation of Attention: A Graph-Based Study

**DOI:** 10.3389/fnint.2019.00067

**Published:** 2019-11-19

**Authors:** Yang Xi, Qi Li, Mengchao Zhang, Lin Liu, Guangjian Li, Weihong Lin, Jinglong Wu

**Affiliations:** ^1^School of Computer Science and Technology, Changchun University of Science and Technology, Changchun, China; ^2^School of Computer Science, Northeast Electric Power University, Jilin, China; ^3^Department of Radiology, China-Japan Union Hospital of Jilin University, Changchun, China; ^4^Department of Neurology, The First Hospital of Jilin University, Changchun, China; ^5^Graduate School of Natural Science and Technology, Okayama University, Okayama, Japan

**Keywords:** semantics, audiovisual stimulus, functional magnetic resonance imaging, functional connectivity, brain network, graph theory

## Abstract

Semantic audiovisual stimuli have a facilitatory effect on behavioral performance and influence the integration of multisensory inputs across sensory modalities. Many neuroimaging and electrophysiological studies investigated the neural mechanisms of multisensory semantic processing and reported that attention modulates the response to multisensory semantic inputs. In the present study, we designed an functional magnetic resonance imaging (fMRI) experiment of semantic discrimination using the unimodal auditory, unimodal visual and bimodal audiovisual stimuli with semantic information. By manipulating the stimuli present on attended and unattended position, we recorded the task-related fMRI data corresponding to the unimodal auditory, unimodal visual and bimodal audiovisual stimuli in attended and unattended conditions. We also recorded the fMRI data in resting state. Then the fMRI method was used together with a graph theoretical analysis to construct the functional brain networks in task-related and resting states and quantitatively characterize the topological network properties. The aim of our present study is to explore the characteristics of functional brain networks that process semantic audiovisual stimuli in attended and unattended conditions, revealing the neural mechanism of multisensory processing and the modulation of attention. The behavioral results showed that the audiovisual stimulus presented simultaneously promoted the performance of semantic discrimination task. And the analyses of network properties showed that compared with the resting-state condition, the functional networks of processing semantic audiovisual stimuli (both in attended and unattended conditions) had greater small-worldness, global efficiency, and lower clustering coefficient, characteristic path length, global efficiency and hierarchy. In addition, the hubs were concentrated in the bilateral temporal lobes, especially in the anterior temporal lobes (ATLs), which were positively correlated to reaction time (RT). Moreover, attention significantly altered the degree of small-worldness and the distribution of hubs in the functional network for processing semantic audiovisual stimuli. Our findings suggest that the topological structure of the functional brain network for processing semantic audiovisual stimulus is modulated by attention, and has the characteristics of high efficiency and low wiring cost, which maintains an optimized balance between functional segregation and integration for multisensory processing efficiently.

## Introduction

Successful human communication critically depends on efficient semantic comprehension, which refers to a collection of interactive cognitive mechanisms that support semantically derived behaviors (Lambon Ralph, 2014). Many studies have demonstrated that compared to unisensory stimulation, semantic audiovisual pairings significantly promote behavioral performances, resulting in faster reaction times (RTs) and higher accuracy (Naghavi et al., 2011; Hu et al., 2012). A substantial body of work has been done on the multisensory semantic processing of audiovisual stimuli (Noesselt et al., 2010; Plank et al., 2012; Ye et al., 2017). Neuroimaging studies have reported that the bilateral superior temporal regions together with the medial prefrontal cortex are strongly activated by semantic speech simultaneously presented with lip movements (Ye et al., 2017). The anterior temporal lobe (ATL) was also found to be activated by semantically matched audiovisual stimuli (Lambon Ralph et al., 2010, 2017; Jackson et al., 2015). Electrophysiological studies revealed that event-related potentials for semantic audiovisual stimuli differ from those for the sum of their unisensory constituents at the late stage of 236–530 ms over the frontal and parietal-occipital electrodes (Xie et al., 2017). Moreover, the event-related potential components related to semantic audiovisual stimuli in attended and unattended conditions have different temporal and spatial distributions (Donohue et al., 2011; Wang et al., 2016). These studies adopted direct or indirect methods to measure cerebral responses to semantic audiovisual stimuli to explore the neural mechanisms that process the bimodal audiovisual stimuli with congruent semantic information, as well as the effects of attention.

Owing to the recent development of connectomics, many studies propose that brain functions are subserved by interactions of large-scale distributed and parallel subnetworks (Catani et al., 2007; de Benedictis and Duffau, 2011), in which the functional connectivity among brain areas is the basis for recognition task processing (Erika-Florence et al., 2014; Hampshire and Sharp, 2015). Cognitive and clinical neuroscience theories generally suggest that the pattern of information processing between neural regions should vary depending on the context or the cognitive process being performed (Botvinick et al., 2001). Thus, the topologies of network connections are specialized and dynamically responsive to task demands (Elbich et al., 2019), and they reflect the interaction patterns underlying information processing in different cognitive tasks (Hampshire and Sharp, 2015; Spielberg et al., 2015). Similarly, functional connectivity exists also in the resting state of the brain. The comparison between the network architectures of the task and the resting state showed that the brain’s functional network architecture during task performance is shaped primarily by an intrinsic network architecture which is also present at rest, and secondarily by evoked task-general and task-specific network changes (Fox and Raichle, 2007; Vincent et al., 2007; Cole et al., 2014). According to the above, we can examine the characteristics of functional brain networks processing bimodal audiovisual stimuli with semantic information to explore the neural mechanisms of multisensory processing.

The characteristics of functional brain networks can be analyzed using an approach based on graph theory. This approach has been previously applied to study network properties in various cognitive tasks (Cao et al., 2013; Cole et al., 2013). Based on graph theory, the human brain can be modeled as a complex network represented graphically by a collection of nodes and edges (Wang et al., 2010; Smith et al., 2013; van den Heuvel and Sporns, 2013; Zuo and Xing, 2014). The nodes are achieved by parceling the human brain into tens to hundreds of small regions (Craddock et al., 2013), and connectivity measurements are then calculated as the strength of the edges between the nodes of this network (Fornito et al., 2013). The global and local topological features of this network support the functional segregation and integration of the brain for cognitive processes (Deco et al., 2015). The analysis method combining functional magnetic resonance imaging (fMRI) with graph theory (Smith et al., 2013; van den Heuvel and Sporns, 2013; Zuo and Xing, 2014) has become recently a powerful tool to study the human brain functions of segregation and integration, and it can provide concise metrics representing the overall functional network organization in response to contextual changes (Bullmore and Sporns, 2009; Godwin et al., 2015).

In the present study, we designed an fMRI experiment of semantic discrimination using the unimodal auditory, unimodal visual and bimodal audiovisual stimuli with semantic information. By manipulating the stimuli present on attended and unattended position, we recorded the task-related fMRI data corresponding to the unimodal auditory, unimodal visual and bimodal audiovisual stimuli in attended and unattended conditions. We also recorded the fMRI data in resting state. Then the fMRI method was used together with a graph theoretical analysis to construct the functional brain networks in task-related and resting states and quantitatively characterize the topological network properties. The global properties, local properties and the correlation between nodal efficiency and RT were calculated and analyzed. The aim of our present study is to explore the characteristics of functional brain networks that process semantic audiovisual stimuli in attended and unattended conditions, and provide new insights into the neural mechanisms of multisensory semantic processing and the modulation of attention in the human brain from a systemic perspective (Zhou et al., 2012; Zuo et al., 2012; Horn et al., 2014).

## Materials and Methods

### Participants

Eighteen healthy volunteers (9 females; age range: 21–27 years, mean age: 24 years) participated in this fMRI study. All subjects were right-handed, and had normal or corrected-to-normal vision (wearing non-metallic glasses during fMRI scans), and reported normal hearing. The Experimental protocol was approved by the Ethics Committee of Changchun University of Science and Technology. After receiving a full explanation of the experimental purpose and the general attention of fMRI scanning, participants gave written informed consent for all experiments as per the protocol approved by the institutional research review board.

### Stimuli

The task utilized unimodal visual (V), unimodal auditory (A), and bimodal audiovisual (AV) stimuli. Visual stimuli were gray-scale pictures of the inanimate (e.g., bell, guitar, car, and clock) or animate objects (e.g., cat, dog, cow, and frog) from the international common Snodgrass-Vanderwart white–black line graphic library and were processed using AI illustrator software (Adobe Systems Inc., San Jose, CA, United States). And auditory stimuli were the sounds corresponding to the inanimate or animate objects obtained from the Internet and were processed using Cool Edit Pro 2.1 software (Adobe Systems Inc., San Jose, CA, United States). Stimulus presentation was controlled by a personal computer running Presentation 0.71 software (Neurobehavioral Systems Inc., Albany, CA, United States).

**FIGURE 1:**
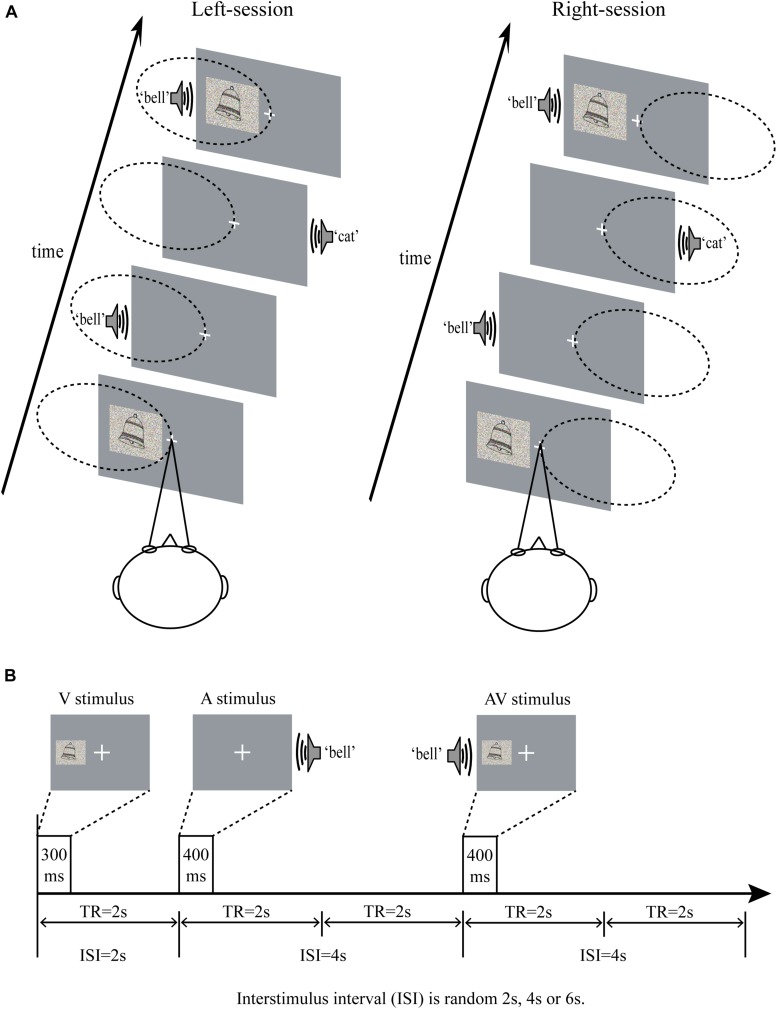
**(A)** Schematic of the experimental design in left-sessions and right-sessions. The participants were instructed to look at the central fixation (a cross), and attended to the stimuli presented on the left side in left-sessions, and attended to the stimuli presented on the right side in right-sessions. The dotted ellipse indicated the position attended to. **(B)** Time course of the stimuli presentation. ISI, interstimulus interval; TR, repetition time; A, auditory; V, visual; AV, audiovisual.

To ensure that the participants focused on the stimuli during the experiment, the stimuli should be presented for as short a time as possible. At the same time, in order to enable the participants to obtain semantic information to discriminate the category (inanimate or animate objects), the stimuli should be presented long enough. Prior to the formal experiment, the appropriate presentation time for pictures and sounds was examined in a behavioral pre-experiment, in which the pictures and sounds were designed to presented for 200, 300, and 400 ms. The participants were required to discriminate the stimuli to be inanimate or animate objects. A minimum presentation time with an across-subject accuracy of above 80% was selected. As a result, the pictures and sounds were determined to be presented 300 and 400 ms, respectively. The visual stimuli (6.0 cm × 4.8 cm, subtending a visual angle of approximately 4.3°, 300 ms duration) were projected onto a screen placed behind the head of the participants at the end of the scanner bore, visible to participants by a mirror placed within the MR head coil. A constant distance of 80 cm was maintained between the eye and the projection screen. As shown in Figure 1A, all the visual stimuli were presented on the left or right side of the display, and the center of the visual stimuli was at an angle of approximately 6° from a centrally presented fixation point (a cross) located directly from the participants’ eyes. While the auditory stimuli were presented in the left or right ear through earphones (44 kHz sampling rate, 400 ms duration, 10 ms rise and fall periods, approximate 80 dB). The interstimulus interval (ISI) varied randomly 2 s, 4 s, and 6 s (Figure 1B). And audiovisual stimuli were the picture and sound that began to appear simultaneously on the ipsilateral side. To appropriately increase the difficulty of the task and maintain participants’ attention, both auditory and visual components were added random noise, making the stimuli presented in a degraded manner (Stevenson and James, 2009; Werner and Noppeney, 2010). The visual stimuli were degraded by weighted averaging the original pictures with random noise images of the identical size. Similar to the procedure for degrading visual stimuli, auditory stimuli were a weighted average of the original sound with a random noise sound of identical length.

### Procedure

An event-related fMRI design was adopted to measure the responses of the brain during a semantic discrimination task. Before the formal experiment, each participant was required to continue training for adapting to the experimental environment and being familiar with the experimental equipment. When accuracy rate reached 80%, the experimenter was convinced that they understood the task. Eight sessions need to be completed for each participant, and each session consisted of 40 A stimuli, 40 V stimuli and 40 AV stimuli. The frequencies of the inanimate and animate stimuli were both 50% for each group of A, V, and AV stimuli. There were six types of stimuli (2 (inanimate and animate objects) × 3 (A, V, and AV)) presented with equal probability on the left and right sides of the participant according to a pseudorandom sequence.

During the experiment, participants were instructed to minimize blinking and bodily movements to avoid movement artifacts. As shown in Figure 1A, the participants were required to fix their eyes on a centrally presented fixation point and to attend to the visual stimuli on one side of the screen and the auditory stimuli presented by the ipsilateral ear, while ignoring the visual stimuli on the opposite side of the screen and the auditory stimuli presented by the other ear. Participants were required to attend to left side in four of eight sessions (named as left-session), and to attend to right side in the other four sessions (named as right-session). The two types of sessions were conducted in an alternating fashion. The task was to press left button when hearing or/and seeing the animate stimuli and press right button when hearing or/and seeing the inanimate stimuli on attended side, with responding as quickly and accurately as possible. All participants were allowed to take a 5-min break between sessions. The AV stimulus that presented on left side in left-sessions and that presented on right side in right-sessions was attended AV. In contrast, the AV stimulus that presented on left side in right-sessions and that presented on right side in left-sessions was unattended AV.

### Behavioral Analysis

Mean RTs, hit rates (HRs), and false alarm rates (FARs) for the target stimuli were computed separately for each stimulus type (AV, A, and V) and location (left and right). In addition, signal sensitivity measures *d*′ = *Z*(HRs) − *Z*(FARs) was also calculated separately for each stimulus type (AV, A, and V) and location (left and right) using signal detection theory (SDT) (Stanislaw and Todorov, 1999). The *Z* is the inverse of the normal cumulative distribution function. Then all the behavioral data was entered into separate repeated-measure analysis of variances (ANOVA) with Type (AV, A, and V) and Location (left and right) as within-subject factors. All statistical analyses for behavioral data were conducted using IBM SPSS software (version 22, IBM Inc., United States) for Windows. Greenhouse–Geisser corrections were applied with adjusted degrees of freedom. A value of *p* < 0.05 was considered to indicate a statistically significant difference. A power analysis was also implemented using GPower3.1 to supported the number of participants, and the value of power >75% was considered to indicate a statistically significant difference.

### Acquisition of fMRI Data

Before audiovisual semantic discrimination experiment, a 5-min resting-state scan was obtained, during which each participant was instructed to rest and focus on the fixation point presented on the central of the screen. All task-state (semantic discrimination task) and resting-state functional imaging data were acquired at the Sino Japanese Friendship Hospital of Jilin University. A 3T fMRI scanner (Siemens) was used to acquire both T1-weighted anatomical images (repetition time (TR) = 8600 ms; echo time (TE) = 4 ms; field-of-view (FOV) = 192 mm; flip angle (FA) = 90°; 128 slices; voxel size = 1 × 1 × 1 mm) and T2-weighted gradient echo planar imaging sequence (TR = 2 s; TE = 30 ms; FOV = 192 mm; FA = 90°; 33 slices; voxel size = 3 × 3 × 3 mm). There were eight sessions with a total of 160 volume images per session. The high-resolution anatomical image volume was acquired at the end of the experiment.

### fMRI Data Preprocessing

The preprocessing of the task-state data during semantic discrimination was conducted using the SPM8 software package (Wellcome Department of Cognitive Neurology, London, United Kingdom) under Matlab2012 (MathWorks, Inc., Natick, MA, United States). For each run, the first six time points were discarded to account for signal equilibrium and the participant’s adaptation to the circumstances. All images were realigned to the first image volume in the series, normalized to standard anatomical space defined by the Montreal Neurological Institute atlas, and smoothed using a 6.0-mm full-width half-maximum Gaussian kernel.

The resting-state data were preprocessed using the SPM8 software package and the Data Processing Assistant for Resting-State fMRI (DPARSF)^1^. After discarding the first six time points, the remaining functional images were realigned to the first volume to correct for head motion and then spatially normalized to standard anatomical space defined by the Montreal Neurological Institute atlas. Temporal bandpass filtering (0.01 ≤ *f* ≤ 0.1 Hz) was performed afterward to reduce the effects of low-frequency drift and high-frequency noise. To reduce the effects of motion and non-neuronal blood oxygen level-dependent fluctuations, the head motion, cerebrospinal fluid signal, and white matter signals were further removed as nuisance covariates (Seeman et al., 2005). Finally, the fMRI images were smoothed using a Gaussian filter with a full width at half maximum of 4 mm.

### Construction of Functional Brain Networks

The task-state fMRI data were entered into the CONN toolbox (Gabrieli Laboratory, Cambridge, MA, United States) to construct individual functional brain networks corresponding to the attended A, attended V, attended AV, unattended A, unattended V, and unattended AV conditions. And the resting-state fMRI data was also entered into the CONN toolbox to construct functional brain network of resting-state condition. The regions of interests (ROIs) were defined based on the anatomical automatic labeling (AAL) (Kelly et al., 2008), which contains 90 regions of the cerebrum and 26 regions of the cerebellum. The group-average correlations of ROI-to-ROI were computed for each participant’s attended A, attended V, attended AV, unattended A, unattended V, unattended AV, and resting-state conditions to construct 116-node whole-brain functional connectivity networks. Seven 116 × 116 correlation matrices were obtained for each subject. False positive control in this ROI-to-ROI analysis is implemented by using *p*-values corrected for the false discovery rate in the CONN toolbox (Whitfield-Gabrieli and Nieto-Castanon, 2012). Overall, brain networks were constructed with a defined sparsity. The sparsity threshold ensures that all resultant networks have comparable topological structures with the same number of edges (Wang et al., 2011). Hence, we selected the equal-interval sparsity threshold range (ranging from 0.05 to 0.5 with a partition interval of 0.05), and individual brain networks were constructed at the same sparsity level across subjects.

### Network Analysis

Graph theoretical analyses were performed using the GRETNA toolbox^2^. The network architecture was investigated at both global and local levels of each functional brain network corresponding to attended A, attended V, attended AV, unattended A, unattended V, unattended AV, and resting-state conditions. First, we compared the global topological properties of bimodal AV network with those of unimodal A, V networks both in attended and unattended conditions using paired *t*-test. And then an ANOVA with the factor of Condition was conducted to compare the global properties in attended AV, unattended AV, and resting-state conditions. We further compared the attended and unattended AV conditions using paired *t*-test for exploring the modulatory effects of attention to the brain network architecture. The global network metrics, including the characteristic path length Lp, clustering coefficient Cp, normalized characteristic path length λ, normalized clustering coefficient γ, small-worldness σ, local efficiency Eloc, global efficiency Eg, assortativity, and hierarchy, were calculated. Small-worldness was originally proposed by Watts and Strogatz (1998) and has a higher local clustering with an equivalent characteristic path length in comparison to random networks (Tzourio-Mazoyer et al., 2002). The topological properties Lp, λ, and Eg are considered as measures of functional integration, whereas the ability of a network’s segregation and error tolerance can be expressed as Cp, γ, and Eloc (Latora and Marchiori, 2001; Sporns and Zwi, 2004). In addition, the nodal degree and efficiency were also calculated to examine the local characteristics of each cortical region in the functional networks of attended and unattended AV conditions (Watts and Strogatz, 1998), determining the important regions for processing bimodal AV stimulus. Moreover, we computed Pearson correlations to analyze the associations between nodal efficiency and RT in the functional network of the attended AV condition, further investigating the influence of local efficiency on behavioral performance.

All statistical analyses were performed using IBM SPSS software (version 22, IBM Inc., United States) for Windows. The network metrics were calculated under ten network sparsity thresholds from 0.05 to 0.5, and the average values of the area under the curve were used for the statistical analysis in order to provide a scalar that did not depend on the specific threshold selection. A value of *p* < 0.05 was considered to indicate a statistically significant difference.

## Results

### Behavioral Result

We analyzed the behavioral data to bimodal AV, unimodal A and V stimuli to examine whether the simultaneous auditory and visual stimuli facilitated the behavioral performance. The results showed no significant differences in mean RTs (*F* = 1.724, *p* = 0.206, power = 99.99%), HRs (*F* = 0.050, *p* = 0.826, power = 99.38%), FARs (*F* = 2.461, *p* = 0.067, power = 94.91%), and *d*′ (*F* = 0.515, *p* = 0.482, power = 99.99%) based on stimulus location. Therefore, the RTs, HRs, FARs and *d*′ to stimuli on the left and right sides were combined to increase the signal-to-noise ratio.

**FIGURE 2:**
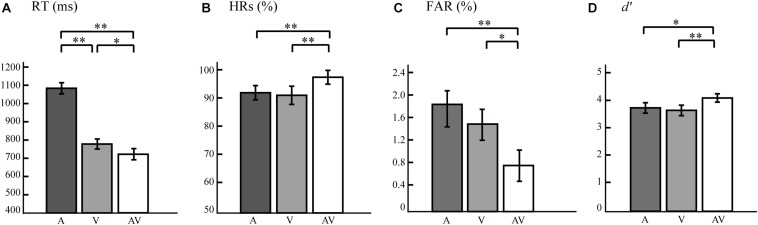
Bar plots showing behavioral results. **(A)** Mean response times (RTs) for A, V, and AV target stimuli. **(B)** Mean hit rates (HRs) for A, V, and AV target stimuli. **(C)** False alarm rates (FARs) for A, V, and AV target stimuli. **(D)** Perception sensitivity (*d*′) for A, V, and AV target stimuli. The error bars represent the SEM. SEM, standard error of mean. ^∗∗^*p* < 0.01, ^∗^*p* < 0.05.

We observed a main effect of Type, verifying that RTs to V, A, and AV stimuli differed significantly from one another (*F* = 507.029, *p* < 0.0005, power = 99.99%). Subsequent paired *t*-tests revealed that participants responded faster to target AV stimuli than to target A stimuli (*t* = 26.199, *p* < 0.0005) and V stimuli (*t* = 2.353, *p* = 0.048). RTs to V target stimuli were also faster than those to A target stimuli (*t* = 21.206, *p* < 0.0005) (Figure 2A). A significant main effect of Type was also found in HRs (*F* = 3.344, *p* < 0.047, power = 98.23%). Paired *t*-tests revealed that HRs to AV was significantly higher than to A target stimuli (*t* = 3.336, *p* = 0.004) and to V target stimuli (*t* = 2.255, *p* = 0.037), but no significant difference was found between the HRs to A and V stimuli (*t* = 0.623, *p* = 0.541; Figure 2B). FARs to target AV, A, and V stimuli were significantly different (*F* = 11.670, *p* < 0.0005, power = 82.55%). Paired *t*-tests showed that the FARs to target AV were significantly lower than that to target A (*t* = 4.752, *p* < 0.0005) and target V stimuli (*t* = 2.840, *p* = 0.011). And the FARs to target A and V stimuli were no significant difference observed (*t* = 1.884, *p* = 0.076; Figure 2C). As shown in Figure 2D, a repeated-measures ANOVA on *d*′ values showed a significant effect of Type (*F* = 4.432, *p* = 0.022, power = 99.95%). Paired *t*-tests showed that *d*′ values for AV target stimuli were significantly higher than those for V target stimuli (*t* = 3.050, *p* = 0.007) and A target stimuli (*t* = 2.407, *p* = 0.027). These results indicated that the bimodal AV stimulus presented simultaneously enhanced the accuracy and sensitivity of semantic discrimination, and reduced the response time and error rate. Thus, we believed that the participants effectively integrated visual and auditory information in this semantic discrimination task.

### Global Properties

**FIGURE 3:**
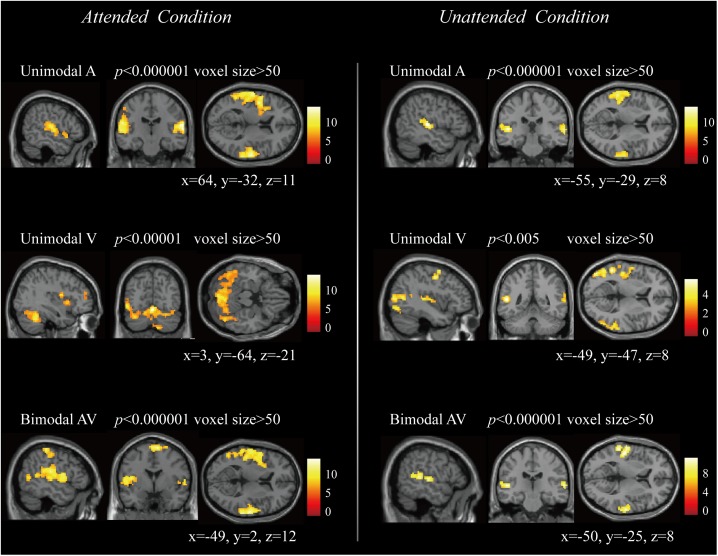
The brain activation to unimodal A, unimodal V, and bimodal AV stimuli in attended and unattended conditions, respectively.

Figure 3 showed the activation results in attended and unattended conditions, including the brain response to unimodal A, unimodal V, and bimodal AV stimuli. These results indicated that the unimodal A and V stimuli activated the primary auditory and visual cortices, and the bimodal AV stimulus activated the bilateral temporal and frontal regions. To explore the architecture of the functional brain network that processes semantic audiovisual stimuli underlying the modulation of attention, we computed the correlations of 116 regions to construct functional connectivities in attended A, attended V, attended AV, unattended A, unattended V, unattended AV, and resting-state conditions, and then compared the global properties between bimodal AV and unimodal A/V conditions, and between the task- and resting-state conditions.

As shown in Table 1, for both attend and unattended conditions, the values of Cp, Eg, and Eloc in the bimodal AV network were significantly greater than those in the unimodal A (Cp: *t* = 2.985, *p* = 0.009; Eg: *t* = 5.409, *p* < 0.0005; Eloc: *t* = 4.652, *p* < 0.0005 in attended condition; Cp: *t* = 4.502, *p* < 0.0005; Eg: *t* = 6.911, *p* < 0.0005; Eloc: *t* = 7.112, *p* < 0.0005 in unattended condition) and V (Cp: *t* = 2.537, *p* = 0.022; Eg: *t* = 4.237, *p* = 0.001; Eloc: *t* = 4.365, *p* < 0.0005 in attended condition; Cp: *t* = 3.693, *p* = 0.002; Eg: *t* = 8.212, *p* < 0.0005; Eloc: *t* = 5.413, *p* < 0.0005 in unattended condition) networks. In addition, for both attend and unattended conditions, the values of Lp in the bimodal AV networks were significantly lower than those in the unimodal A (*t* = −4.883, *p* < 0.0005 in attended condition; *t* = −5.729, *p* < 0.0005 in unattended condition) and V (*t* = −3.755, *p* = 0.002 in attended condition; *t* = −7.911, *p* < 0.0005 in unattended condition) networks. The greater global efficiency, local efficiency, clustering coefficient and the lower characteristic path length revealed that the brain has a greater capacity of functional integration (greater Eg and lower Lp in AV condition) and local processing (greater Cp and Eloc in AV condition) during bimodal audiovisual processing (Latora and Marchiori, 2001; Sporns and Zwi, 2004). In addition, for the attended condition, the value of γ in bimodal AV network was smaller than those in unimodal A (*t* = −2.331, *p* = 0.033) and V (*t* = −3.204, *p* = 0.006) networks, and the value of Lp in bimodal AV network was smaller than that in unimodal A network (*t* = −4.883, *p* < 0.0005). While for the unattended condition, the value of λ in bimodal AV network was smaller than that in unimodal V network (*t* = −7.911, *p* < 0.0005). There was no other significant difference between bimodal AV network and unimodal A/V network in attended and unattended conditions.

**TABLE 1:** The analysis results of paired *t*-test between bimodal AV and unimodal A/V networks in attended and unattended conditions respectively.

	**Attended condition**		**Unattended condition**	
	**AV and A**	**AV and V**	**AV and A**	**AV and V**
Cp	*t* = 2.985, *p* = 0.009^∗∗^	*t* = 2.537, *p* = 0.022^∗^	*t* = 4.502, *p* < 0.0005^∗∗^	*t* = 3.693, *p* = 0.002^∗∗^
Gamma	*t* = −0.028, *p* = 0.756	*t* = −0.468, *p* = 0.703	*t* = 0.016, *p* = 0.988	*t* = −0.515, *p* = 0.613
Lambda	*t* = −1.224, *p* = 0.231	*t* = −0.793, *p* = 0.439	*t* = −1.867, *p* = 0.080	*t* = −1.145, *p* = 0.289
Lp	*t* = −4.883, *p* < 0.0005^∗∗^	*t* = −3.755, *p* = 0.002^∗∗^	*t* = −5.729, *p* < 0.0005^∗∗^	*t* = −7.911, *p* < 0.0005^∗∗^
Sigma	*t* = −2.060, *p* = 0.078	*t* = −1.681, *p* = 0.112	*t* = 0.694, *p* = 0.498	*t* = 0.397, *p* = 0.697
Eg	*t* = 5.409, *p* < 0.0005^∗∗^	*t* = 4.237, *p* = 0.001^∗∗^	*t* = 6.911, *p* < 0.0005^∗∗^	*t* = 8.212, *p* < 0.0005^∗∗^
Eloc	*t* = 4.652, *p* < 0.0005^∗∗^	*t* = 4.365, *p* < 0.0005^∗∗^	*t* = 7.112, *p* < 0.0005^∗∗^	*t* = 5.413, *p* < 0.0005^∗∗^
Hierarchy	*t* = −0.373, *p* = 0.714	*t* = −0.220, *p* = 0.829	*t* = 1.388, *p* = 0.184	*t* = 0.725, *p* = 0.479
Assortativity	*t* = 0.531, *p* = 0.602	*t* = −0.056, *p* = 0.956	*t* = −0.322, *p* = 0.752	*t* = −0.626, *p* = 0.540

*^∗^*p* < 0.05, ^∗∗^*p* < 0.01.*

**FIGURE 4:**
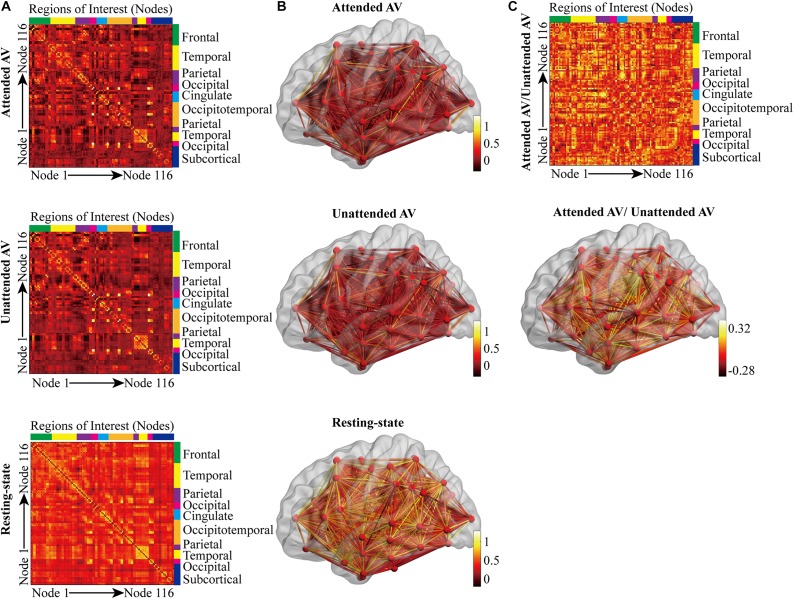
Correlation matrices and connectivity patterns of the functional brain networks in three conditions. **(A)** The correlation matrices calculated based on 116 regions in the anatomical automatic labeling (AAL) template, corresponding to the attended AV, unattended AV, and resting-state conditions. **(B)** The subject-averaged functional connectivity patterns in the brain plotted for the attended AV, unattended AV, and resting-state conditions. The color bar indicates the functional connection strength. **(C)** Differences between subject-averaged connectivity patterns plotted for the attended AV minus the unattended AV condition. Brighter colors depict stronger connections for the attended AV than for the unattended AV condition, whereas darker colors indicate that the unattended AV connections are stronger than the attended connections.

**FIGURE 5:**
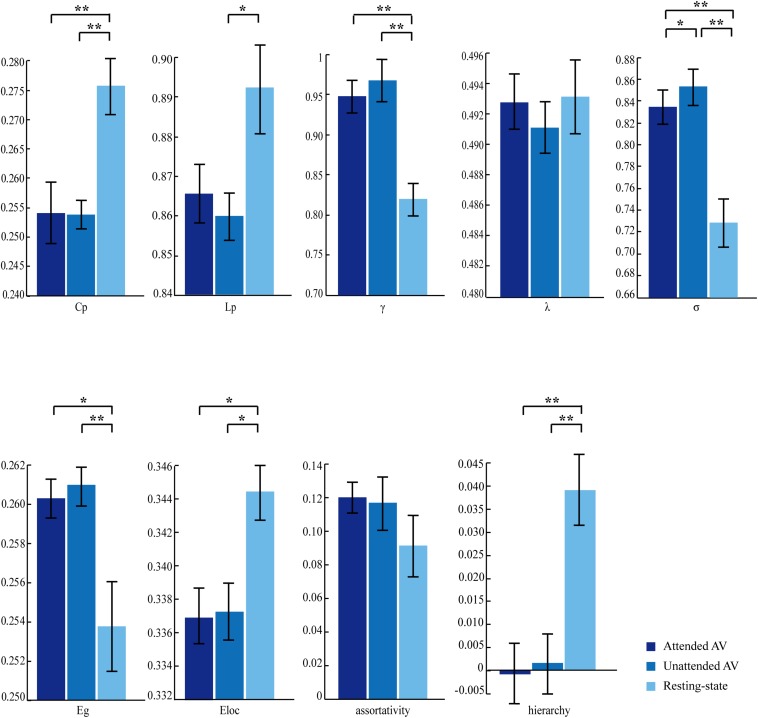
Effects of the attended AV, unattended AV, and resting-state conditions on the graph theory metrics of global properties. Cp, clustering coefficient; Lp, characteristic path length; γ, normalized clustering coefficient; λ, normalized characteristic path length; σ, small-worldness; Eg, global efficiency; Eloc, local efficiency. ^∗^*p* < 0.05, ^∗∗^*p* < 0.01.

The three correlation matrices and the functional connectivity patterns corresponding to the attended AV, unattended AV and resting-state conditions averaged across all participants were descripted in Figures 4A,B. We further subtracted the correlation matrix of unattended AV condition from that of attended AV condition, and got the functional connectivity pattern in the brain, reflecting the modulatory effect of attention (Figure 4C). Based on these correlation matrices, we calculated and compared the global properties of these three networks. The ANOVA analysis showed a main effect of the Condition (attended AV, unattended AV, and resting-state conditions) on the network properties Cp (*F* = 41.540, *p* < 0.0005, power = 99.98%), Lp (*F* = 4.081, *p* = 0.048, power = 79.56%), γ (*F* = 16.126, *p* = 0.001, power = 99.98%), σ (*F* = 15.150, *p* = 0.001, power = 99.96%), Eg (*F* = 7.995, *p* = 0.011, power = 99.98%), Eloc (*F* = 7.568, *p* = 0.011, power = 90.06%), and hierarchy (*F* = 8.304, *p* = 0.002, power = 88.14%). However, no significant main effects of Condition were found in λ (*F* = 0.342, *p* = 0.617, power = 7.995%) and assortativity (*F* = 2.913, *p* = 0.085, power = 46.08%). These results revealed that the architectures of the functional network in the brain were altered depending on the current task (processing of attended or unattended semantic AV stimuli or being at rest). As shown in Figure 5, paired *t*-tests showed that there were significant differences in Cp (*t* = −7.288, *p* < 0.0005), γ (*t* = 3.597, *p* = 0.003), σ (*t* = 3.369, *p* = 0.004), Eloc (*t* = −2.814, *p* = 0.013), Eg (*t* = 2.597, *p* = 0.02), and hierarchy (*t* = −3.184, *p* = 0.006) between the functional networks of attended AV and resting-state conditions, revealing that the brain network had lower clustering coefficient, local efficiency and greater global efficiency. Similar significant differences were obtained between the functional networks of unattended AV and resting-state conditions in Cp (*t* = −6.440, *p* < 0.0005), Lp (*t* = −2.557, *p* = 0.022), γ (*t* = 4.605, *p* < 0.0005), σ (*t* = 4.639, *p* < 0.0005), Eloc (*t* = −2.811, *p* = 0.013), Eg (*t* = 3.071, *p* = 0.008), and hierarchy (*t* = −3.684, *p* = 0.002). In addition, a significant difference was also observed in σ (*t* = −2.173, *p* = 0.046) between the functional networks of attended and unattended AV conditions.

### Nodal Properties

We calculated the node degree and efficiency of the functional brain networks in attended and unattended AV conditions to identify the nodes whose value of degree (or efficiency) were larger than the sum of average value and standard deviation across all nodes of the network as hub nodes.

**FIGURE 6:**
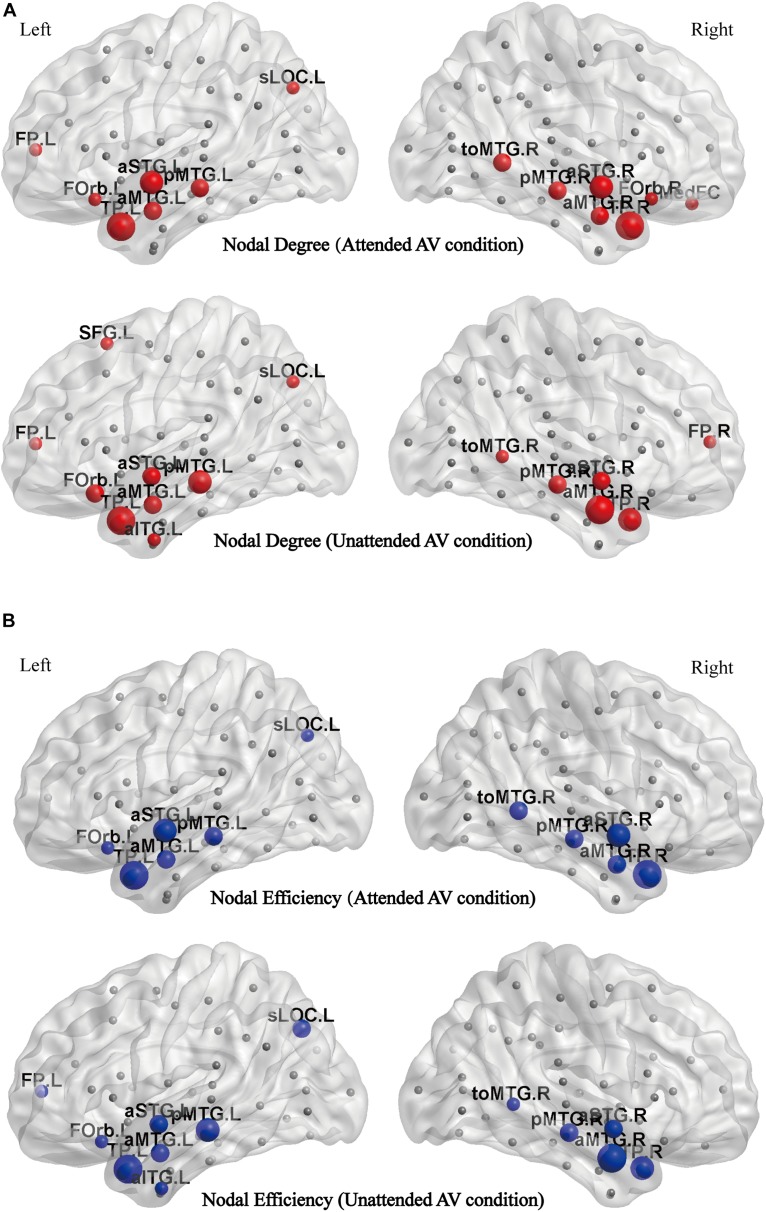
**(A)** The distribution of hubs with a high nodal degree in the attended and unattended AV conditions mapped to the cortical surface. **(B)** The distribution of hubs with a high nodal efficiency in the attended and unattended AV conditions mapped to the cortical surface. aITG, inferior temporal gyrus; aMTG, anterior middle temporal gyrus; aSTG, anterior superior temporal gyrus; FOrb, frontal orbital cortex; FP, frontal pol; MedFC, medial frontal cortex; pMTG, posterior middle temporal gyrus; sLOC, superior lateral occipital cortex; SFG, superior frontal gyrus; toMTG, temporo-occipital middle temporal gyrus; TP, temporal pole; L, left; R, right.

The distributions of the degree hubs as described in Figure 6A show that twelve common hubs were shared in functional networks corresponding to the attended AV and unattended AV conditions. These hubs are the left temporal pole (TP.L), right temporal pole (TP.R), left anterior superior temporal gyrus (aSTG.L), right anterior superior temporal gyrus (aSTG.R), left posterior middle temporal gyrus (pMTG.L), right posterior middle temporal gyrus (pMTG.R), right temporo-occipital middle temporal gyrus (toMTG.R), left superior lateral occipital cortex (sLOC.L), left frontal pole (FP.L), left frontal orbital cortex (FOrb.L), left anterior middle temporal gyrus (aMTG.L), and right anterior middle temporal gyrus (aMTG.R). And the medial frontal cortex (MedFC) and right frontal orbital cortex (FOrb.R) are the hubs specific to the attended AV condition, whereas the right frontal pole (FP.R), left superior frontal gyrus (SFG.L), and left anterior temporal gyrus (aITG.L) are specific to the unattended AV condition. Figure 6B shows the distributions of the efficiency hubs in the attended and unattended AV networks. There are 11 hubs with high nodal efficiency in the attended AV networks, including TP.L, TP.R, aSTG.L, aSTG.R, pMTG.L, pMTG.R, toMTG.R, aMTG.L, aMTG.R, sLOC.L, and FOrb.L. These nodes are also the efficiency hubs in the unattended AV networks. And FP.L and aITG.L are two efficiency hubs specific to the unattended AV condition. These results indicated that the hubs with high nodal degree and efficiency are concentrated in the bilateral temporal lobes both in attended and unattended AV networks. In addition, the two nodes with the highest degree and efficiency were TP.L and TP.R in the attended condition.

### Relationship Between Nodal Efficiency and Behavior Performance

**FIGURE 7:**
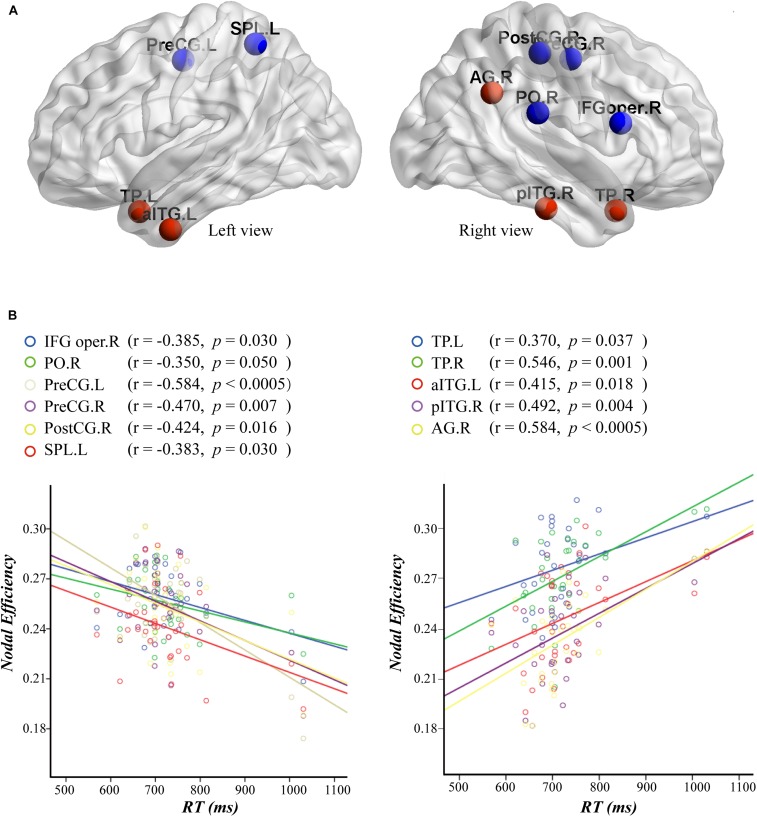
**(A)** Nodes with an efficiency value significantly correlated to the reaction time (RT) are mapped to the cortical surface. The efficiency values of nodes in red and blue are positively and negatively correlated with RT, respectively. **(B)** Scatterplots and robust regression fitting lines for negative (left) and positive (right) correlations between nodal efficiency and RT. The results of correlation analysis and the activation value of the nodes in parentheses. AG, angular gyrus; aITG, anterior inferior temporal gyrus; IFG oper, pars opercularis inferior frontal gyrus; pITG, posterior inferior temporal gyrus; PO, parietal operculum cortex; PostCG, postcentral gyrus; PreCG, precentral gyrus; SPL, superior parietal lobule; TP, temporal pole; L, left; R, right.

Furthermore, we computed a Pearson correlation between nodal efficiency and RT in the attended AV condition. The results are shown in Figure 7. The correlation analysis showed that the nodal efficiencies in the left precentral gyrus (PreCG.L; *r* = −0.584, *p* < 0.0005), right precentral gyrus (PreCG.R; *r* = −0.470, *p* = 0.007), right postcentral gyrus (PostCG.R; *r* = −0.424, *p* = 0.016), right pars opercularis inferior frontal gyrus (IFG oper.R; *r* = −0.385, *p* = 0.030), left superior parietal lobule (SPL.L; *r* = −0.383, *p* = 0.030), and right parietal operculum cortex (PO.R; *r* = −0.350, *p* = 0.050) were significantly negatively correlated with the RT values. Moreover, the nodal efficiencies in the right angular gyrus (AG.R; *r* = 0.584, *p* < 0.0005), TP.R (*r* = 0.546, *p* = 0.001), TP.L (*r* = 0.370, *p* = 0.037), right posterior inferior temporal gyrus (pITG.R; *r* = 0.492, *p* = 0.004), and aITG.L (*r* = 0.415, *p* = 0.018) were significantly positively correlated with the RT values.

## Discussion

The functional brain networks in cognitive tasks and in the resting state have been previously investigated (Congdon et al., 2010; Cole et al., 2013; Erika-Florence et al., 2014; Godwin et al., 2015; Hampshire and Sharp, 2015). However, to the best of our knowledge, our study is the first to investigate the effects of semantic audiovisual stimulus and attention on the architecture of functional brain networks. Using graph theory, we identified the characteristics of functional brain networks for processing bimodal audiovisual stimulus that balance between functional segregation and integration to support semantic discrimination tasks underlying the modulation of attention.

### Global Network Topologies

We compared the global network topologies between bimodal AV and unimodal A/V networks both in the attended and unattended conditions. The results showed greater Cp, Eg, Eloc, and lower Lp for attended and unattended bimodal AV networks, compared with unimodal A/V networks. The greater Cp and Eloc indicated the higher local processing capacity, and the lower Lp and greater Eg indicated the higher global processing capacity. These results suggested that the brain network for processing bimodal auditory stimuli is more optimized, which may be an important reason for bimodal AV stimulus promoting the behavioral performance (Figure 2).

A key finding of the present study is that the σ value for the functional brain network processing semantic audiovisual stimuli is higher than that for the network at resting state (Figure 5), indicating a stronger small-worldness during semantic discrimination task. The small-worldness topology is a fundamental principle of the structural and functional organization of complex brain networks and has greatly impacted studies investigating topological architectures using a systematic perspective (Bullmore and Sporns, 2009; Sporns, 2011). It is commonly agreed that a small-world organization of a network implies both high Cp and low Lp values, reflecting an optimal configuration between the functional segregation and integration of brain networks (Rubinov and Sporns, 2010; Sporns, 2011; Deco et al., 2015; Bassett and Bullmore, 2016). Our finding showed that the values of σ during semantic discrimination task (both in attended and unattended AV conditions) were higher than that at the resting state (Figure 5), indicating that the configuration of the brain network had become more optimized by reinforcing the fine balance between segregation and integration (Rubinov and Sporns, 2010; Deco et al., 2015) when processing a bimodal semantic task. This structure has also been found in functional brain networks executing other tasks. For example, a functional brain network with elevated small-worldness was detected when the participants implemented the color-word Stroop task (Rubinov and Sporns, 2010; Bassett and Bullmore, 2016). We suggest that an increased demand in the task-state condition is linked to a strong small-world pattern which is often observed in networks with optimal configuration for processing (Spielberg et al., 2015), because networks of this type tend to show elevated computational power, low wiring cost, efficient parallel processing, and rapid adaptive reconfiguration (Deco et al., 2015). This interpretation is also consistent with prior evidence that nervous systems are organized to minimize wiring costs (Bassett and Bullmore, 2009).

Importantly, increases in functional integration (higher Eg values), as well as decreases in segregation and specialization (lower Eloc and Cp values), were observed in task-state networks processing semantic audiovisual stimuli compared to those parameters determined in the resting-state condition. The brain regulates information flow by balancing the segregation and integration of incoming stimuli to facilitate flexible cognition and behavior. The topological features of brain networks – in particular, network communities and hubs – support this segregation and integration (Deco et al., 2015). Our results showed that bimodal semantic processing was associated with a restructuring of the global network into a configuration for a more efficient communication across the network (functional integration) with less specialized processing (functional segregation). In our present study, the behavioral data showed that the temporally, spatially, and semantically congruent audiovisual stimuli facilitated semantic discrimination, revealing that the auditory and visual stimuli were integrated, which involves information interaction between multiple cortices across the whole brain (Noesselt et al., 2010; Naghavi et al., 2011; Hu et al., 2012; Plank et al., 2012; Ye et al., 2017). Therefore, we speculate that the higher Eg and lower Eloc values may reflect the specific configuration of the functional brain network for multisensory integration of consistent audiovisual inputs.

In addition, brain networks also typically comprise a hierarchy of communities, and a lower degree of hierarchy was observed in attended AV and unattended AV conditions than in the resting-state condition (Figure 5). The hierarchical organization is evident when high-degree nodes have low clustering, which implies that these nodes act as topological bridges between otherwise unconnected low-degree nodes (Traud et al., 2011). The lower hierarchy in the task-state conditions implied an increase in connections among hub neighbor nodes. In other words, there are more connecting paths between nodes in the functional brain network, in which the communication and interaction of information do not depend on any single connectivity and node. This non-hierarchical structure is considered to be resilient and stable (Traud et al., 2011), providing the basis for the robust transmission of information.

### The Modulation of Attention on the Network Properties

In our study, an increased σ value was observed in the brain network of the unattended AV condition in comparison to that of the attended AV condition (Figure 5), indicating that attention modulated the small-worldness of the brain network for bimodal semantic processing (Latora and Marchiori, 2001; Sporns and Zwi, 2004). Small-world networks can be described by high local clustering and minimum path length, which are reflected by Cp and Lp, respectively (Wang et al., 2010). The analysis showed that there were no statistically significant differences in Cp and Lp between attended and unattended AV conditions, but the relatively lower Lp values produced significantly higher σ values in the brain network of the unattended AV condition. For the unattended AV condition in the present study, the participants were instructed to ignore the stimuli on unattended side and suppress responses, which maybe resulted in a response inhibition consistent with that observed in the go/no-go task (Spielberg et al., 2015). In go/no-go task, a functional brain network with elevated small-worldness was reported to exist in the no-go condition, and the increased response inhibition was linked to its optimal configuration (Spielberg et al., 2015). Thus, we suggest that in the inhibited state a more optimal balance between functional segregation and integration is required than in the response state to reduce wiring costs and increase parallel processing efficiency (Deco et al., 2015).

In addition, attention altered the distributions of the degree and efficiency hubs (Figure 6). The hubs for processing bimodal AV stimulus in attended and unattended conditions were concentrated in the bilateral temporal lobes, especially in the anterior regions, revealing that these nodes had an increased influence on the general network of processing audiovisual inputs. The bilateral TP exhibited the highest degree and efficiency indicating that these regions might contribute more to the efficient information interaction in processing semantic bimodal stimulus, which is consistent with recent studies (Lambon Ralph et al., 2010, 2017; Jackson et al., 2015). Accumulating evidence from functional neuroimaging studies on the ATL in neurologically intact participants found that the ATLs are activated for a range of semantic tasks, irrespective of the input modality (e.g., words, pictures, sounds) (Patterson et al., 2007). Moreover, several neuroscience studies implicate that the convergence of sensory information in the temporal lobe is a graded process that occurs along both its longitudinal and lateral axes and culminates in the most rostral limits (Deco and Rolls, 2004; Rauschecker and Scott, 2009). Furthermore, for multisensory tasks, ATL is considered to contain a high-dimensional modality-independent semantic space that allows computations to be based on semantic information rather than purely sensory similarities, but the same areas do not appear to be involved in equally demanding non-semantic tasks (Lambon Ralph, 2014). These studies provide evidence that the ATL areas are implicated in semantic processing. Interestingly, the two important regions MedFC and SFG are specific to the functional networks in the attended and the unattended AV conditions, respectively. The MedFC is located in the ventromedial frontal lobe, which is considered to play a role in guiding attention to stimulus features associated with the current task (Vaidya and Fellows, 2015). This brain area is also robustly connected with subcortical regions and higher-order sensory regions that provide support for its role in integrating current task-relevant inputs from different modalities (Philiastides et al., 2010; Lim et al., 2013). Therefore, in the present study, the MedFC as a hub specific to the attended AV condition may reflect its central role in the integration of task-relevant audiovisual stimuli with attention. By contrast, the SFG is a hub specific to the unattended AV condition and is considered to participate in cognitive control functions (Zhang et al., 2012). This region with a high nodal degree in the functional network of the unattended condition may reflect the mechanism of cognitive control, which inhibits the participants from responding to the stimuli on the unattended side by pressing a button. However, additional studies are needed to confirm and further elucidate these findings.

### Relationship Between Nodal Efficiency and Reaction Time

We also analyzed the correlation between nodal efficiency and behavioral performance in the attended AV condition The results showed that the efficiencies in PreCG.L, PreCG.R, PostCG.R, IFG oper.R, SPL.L, and PO.R were negatively correlated with the RT (Figure 7), indicating that with the improvement of the information processing efficiency in these nodes, the participants performed the semantic discrimination task more quickly. The nodes SPL.L, PO.R, IFG oper.R, and CO.R are distributed in the frontoparietal and attention networks, which play prominent roles in the allocation and orientation of attention resources (Woldorff et al., 2004; Grent-’t-Jong and Woldorff, 2007). Attention is an essential cognitive function that allows humans and other animals to continuously and dynamically select particularly relevant stimuli from all the available information presented in the external or internal environment so that more neural resources can be devoted to their processing (Li et al., 2010; Talsma et al., 2010). The frontoparietal network of brain areas has been shown to be involved in allocating and controlling the direction of top-down attention by sending control signals that modulate the sensitivity of neurons in sensory brain regions (Woldorff et al., 2004; Grent-’t-Jong and Woldorff, 2007). Moreover, the regions PreCG.R, PreCG.L, and PostCG.R are located in the primary somatosensory and motor cortices (Iwamura, 1998), providing the possibility that the response time is associated with the output of motion in addition to the ability to process input information. In the present study, the participants were asked to focus on the stimuli on the attended side and press the button to determine the semantics, and this cognitive process is related to the cortices dealing with attention and movement. Therefore, we speculate that efficient allocation and orientation of attention resources in brain regions of the frontoparietal network, as well as the efficient processing in the motor cortex to ultimately press the button, facilitated the cognitive processing of semantic discrimination, resulting in a shorter RT.

By contrast, the efficiencies of the nodes AG.R, TP.R, TP.L, pITG.R, and aITG.L distributed in the temporal lobe are positively associated with the RT (Figure 7), indicating that with an improvement of the information processing efficiency in these nodes, the performance for the audiovisual discrimination task was decreased. The regions TP.R and TP.L are located in the bilateral ATLs, which are clearly considered to be central to semantic knowledge (Lewis et al., 2018), especially for the associative relations of semantic multisensory inputs (Jackson et al., 2015). In addition, the AGs can be found in the temporoparietal junction, which has been proposed to link contextually relevant information to the target concept (Schwartz et al., 2011). These studies indicate that these nodes are related to the formation and processing of audiovisual semantic relationships. Moreover, it is generally considered that the RT is sensitive to late-stage cognitive manipulation, reflecting high-level cognitive processes (Van Ede et al., 2012; Li et al., 2017). We suggest that in our experiments, the efficient processing of semantic tasks in these regions consumed a lot of resources and energy in the network and decreased the efficiency of decision-making that occurs at a relatively late cognitive stage, thus resulting in a longer RT. However, further studies are required to confirm this hypothesis.

## Conclusion

In the present study, we tested global and local properties of functional brain networks of processing semantic audiovisual stimulus in attended and unattended conditions, using graph theoretical analysis applied to fMRI data acquired in a semantic discrimination task. Our findings indicate that when process the semantic audiovisual stimulus, the functional network in the brain is optimized to balance the functional segregation and integration for processing the cognitive task with high efficiency and low cost. In addition, attention altered the degree of small-worldness and the distribution of hubs in the functional brain network when processing semantic audiovisual stimuli, revealing the modulatory effect of attention on the multisensory processing from a system perspective. However, one possible limitation is that the functional networks established in this study are undirected, thus lacking information on the directionality of the connections. Therefore, it is likely that some characteristics related to direction are not reflected by our results, such as the modulation of attention to the information interaction in the network. However, these issues can be explored in future studies.

## Data Availability Statement

All datasets generated for this study are included in the article/supplementary material.

## Ethics Statement

The Experimental protocol was approved by the Ethics Committee of Changchun University of Science and Technology. The patients/participants provided their written informed consent to participate in this study.

## Author Contributions

QL, YX, and JW designed the study. QL, YX, MZ, and GL implemented the experiment. YX, WL, and MZ analyzed the data. QL, YX, and LL wrote the manuscript.

## Conflict of Interest

The authors declare that the research was conducted in the absence of any commercial or financial relationships that could be construed as a potential conflict of interest.

## Footnotes

1 http://www.restfmri.net/forum/dparsf

2 http://www.nitrc.org/projects/gretna/
